# Exploring the catalytic potential of transition metal decorated Al_12_N_12_ nanocages as a single atom catalyst for efficient hydrogen adsorption and dissociation reaction

**DOI:** 10.3389/fchem.2026.1879125

**Published:** 2026-09-03

**Authors:** Ali Hussain, Aaiza Saif, Abrar Qadir, Fizza Anjum, Hamna Sharif, Muhammad Sajjad, Abdur Rauf, Mazhar Amjad Gilani, Junaid Yaqoob

**Affiliations:** 1 Department of Chemical and Environmental Engineering, Faculty of Science and Engineering, University of Nottingham, Ningbo, China; 2 Department of Chemistry, University of Okara, Okara, Pakistan; 3 School of Chemistry and Molecular Engineering East China University of Science and Technology, Shanghai, China; 4 Department of Chemistry, University of Sahiwal, Sahiwal, Pakistan; 5 Department of Chemistry, COMSATS University Islamabad, Lahore Campus, Lahore, Pakistan

**Keywords:** Al_12_N_12_ nanocage, activation barrier, adsorption, hydrogen dissociation reaction (HDR), single atom catalysts (SACs), transition metals (TMs)

## Abstract

Hydrogen is a key alternative to fossil fuels and plays a crucial role in industrial hydrogenation. This study employs first-principles density functional theory (DFT) simulations to investigate transition-metal-decorated Al_12_N_12_ single-atom catalysts (SACs) for H_2_ adsorption and dissociation. Interaction energy calculations indicate excellent thermodynamic stability of the catalysts, with Ti@Al_12_N_12_ exhibiting the strongest interaction energy of −2.56 eV. Natural bond orbital (NBO) and frontier molecular orbital (FMO) analyses reveal significant electron transfer from TM atoms to Al_12_N_12_, and a reduction of the HOMO-LUMO gap from 3.86 eV to 1.72 eV. The Ti@Al_12_N_12_ catalyst exhibits the lowest activation barrier, 0.005 eV, for H_2_ dissociation. Mechanistic insights indicate that atomic hydrogen (2H*) is more stable than molecular H_2_, with an energy release up to −1.64 eV. Bidirectional IRC calculations validated the H_2_ dissociation pathways on TM@Al_12_N_12_ catalysts. The blue patches in IRI analysis confirm covalent interactions between hydrogen and TM atoms. This study provides insights into designing efficient SACs for hydrogen dissociation.

## Graphical Abstract

**Figure:**
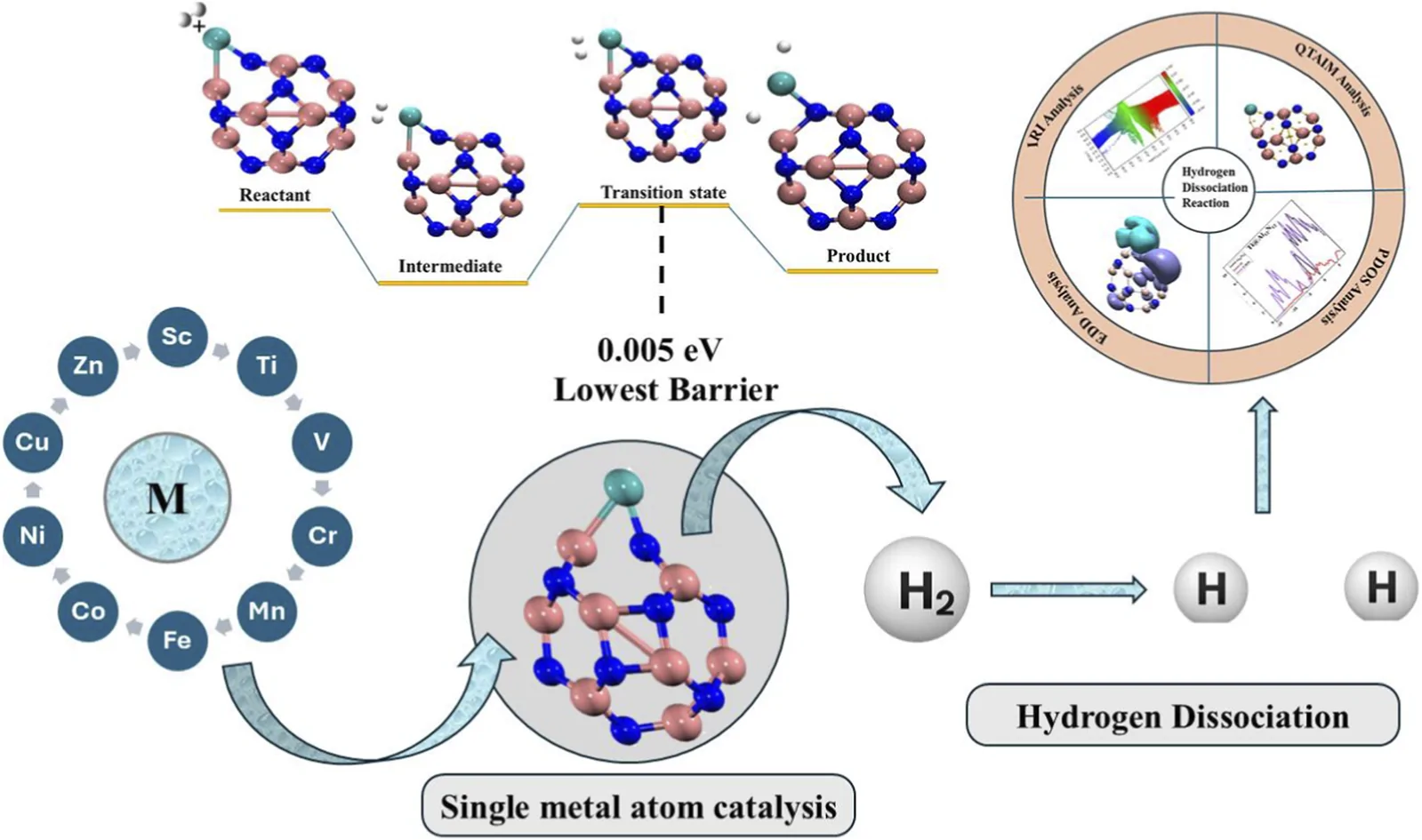

## 1 Introduction

Due to the growing risks of global warming caused by the use of fossil fuels, it is essential for the world to discover new methods for using limitless energy sources (Niemann et al., 2008). The use of non-renewable energy sources must be avoided, as they are making our climate extremely unsafe to live. As global development increases, energy consumption continues to rise. Among authentic alternatives, hydrogen energy is assumed to play a pivotal role in the future and significantly affect various societal advancements. Solar, wind, ocean waves, and hydrogen, which are renewable energy sources, have evolved as the most promising options for meeting the need for energy in the future (Hussain et al., 2024). Hydrogen offers an infinite supply since it can be generated through the electrolysis of water. This process generates hydrogen gas, which, upon combustion, recombines with oxygen to form water (Taneja et al., 2024). The primary byproduct of using hydrogen fuel in fuel cells or combustion processes is water, resulting in negligible environmental impact and almost no greenhouse gas (GHG) emissions (Habib et al., 2024). In addition to its efficiency as a clean fuel, hydrogen has numerous applications. It is extensively used in the production of ammonia, urea, the upgrading of Fischer-Tropsch Gas-to-Liquid (FT-GTL) products, and crude oil refining. In the future, hydrogen is expected to be utilized across various industries, including transportation, gas grids, chemicals, and iron and steel production (Abdin et al., 2020).

Metal hydrides are gaining attention as a better solution for hydrogen storage due to their unique properties, which align well with the requirements for efficient energy systems. The high hydrogen content by weight in these materials enables compact storage, making them suitable for applications ranging from transportation to stationary power systems. Additionally, the cost-effectiveness and scalability of metal hydrides are crucial factors in their potential to support widespread hydrogen adoption. As hydrogen technology continues to evolve, the development of advanced metal hydrides is considered a better alternative to overcome the storage challenges that currently limit the broader implementation of hydrogen as a clean energy source. By addressing these challenges, metal hydrides can significantly contribute to the transition towards more sustainable and environmentally friendly energy systems (Yermakov et al., 2013). Hydrogen dissociation on metal surfaces is a classic model for understanding bond breaking on surfaces. It is studied extensively using both experiments and theories and examined that Porous bifunctional supports improve reactant accessibility and catalytic performance (Feng et al., 2023). Theoretical understanding of this process has been significantly enhanced by more powerful computers and new algorithms (Groß, 1998). Hydrogen technology offers numerous applications, but its practical use still faces challenges. One major obstacle to implementing hydrogen technology effectively is the need for a reliable and affordable method of storing hydrogen. The hydrogen dissociation reaction (HDR) involves splitting molecular hydrogen (H_2_) into atomic hydrogen (2H), a crucial step in hydrogen storage and hydrogenation processes.

The noble metals, such as Ru, Ir (Li and Baek, 2020), Pt, and Pd (Kustov and Kalenchuk, 2022) exhibit excellent catalytic properties for hydrogen dissociation reactions. Noble metals have found extensive use in catalysis because of their distinctive inherent characteristics and vital catalytic capabilities (Zhang et al., 2021). On the other hand, the higher cost and limited availability of the aforementioned metals pose significant challenges. To address these economic concerns in sustainable chemistry, researchers have become increasingly interested in finding alternatives to noble metals for catalytic uses (Konovalova et al., 2023). Scientists have optimized the use of inexpensive, earth-abundant first-row transition metals to efficiently perform complex tasks like small molecule activation and the selective conversion of challenging substrates, making them promising alternatives for catalytic applications (van der Vlugt, 2012). To address the challenges of catalysis, many catalytic methods have been developed. Single-atom catalysts (SACs), which consist of isolated metal atoms decorated on a solid support, have emerged as a highly popular area of research in heterogeneous catalysis (Choudhary et al., 2024). Cu single-atom sites provide selective catalytic activity, demonstrating the effectiveness of isolated metal centers (Liu et al., 2026).

The primary objective of these explorations is to reduce the use of costly and scarce noble metals while enhancing the productivity and selectivity of chemical reactions. SACs represent a major advance in miniaturizing metal-particle technology, in which each metal atom is fully utilized by being dispersed as isolated entities on a support material. This is especially useful when using expensive noble metals. The uniqueness of SACs lies in their uniformity: because each metal atom is isolated and well-defined, these catalysts offer the exciting possibility of achieving extraordinary levels of both activity and selectivity in chemical reactions, making them strikingly promising tool in catalysis (Yang et al., 2013). The catalytic behavior of single-atom catalysts is governed not only by the identity of the isolated metal atom but also by its coordination environment and interaction with the Supplementary Material. Liao et al. demonstrated that the neighboring density of supported single and few-atom sites modifies their local electronic environment and consequently regulates reactant activation and intermediate adsorption–desorption behavior (Liao et al., 2025). Similarly, the cooperative action of Zn–N_3_ single-atom sites and adjacent defective-carbon sites was shown to facilitate sequential hydrogenation steps (Zhai et al., 2026). More broadly, electronic modulation, metal–support interactions, hydrogen spillover and precise active-site engineering have been identified as important design parameters for selective hydrogenation catalysts (Zhou et al., 2026). These findings motivate the present examination of transition-metal-decorated Al_12_N_12_ nanocages, in which the cage can stabilize isolated metal centers while modulating their electronic structure and H_2_-activation ability.

Sarfraz et al. investigated the C20 Fullerene-Based SAC for the H-dissociation reaction. Their findings suggest that Zn@C20 exhibits remarkable catalytic activity due to a low activation energy barrier (Sarfaraz et al., 2023) The Ti-based Mg(0001) surface is also probed for the H-dissociation studies. This study shows that the Ti@Mg(0001) surface is a good one for dissociation of H_2_ with a very low energy barrier of 0.103 eV (Du et al., 2005). Nanobelts decorated with first-row transition metals for H-dissociation were also reported by Bayach et al. (2023). Pt/MoO3 is a highly effective catalyst that achieves nearly complete conversion (99%) and almost perfect selectivity (around 99%) even under ordinary conditions. It is one of the best catalysts known for hydrogenating compounds that contain sulfur atoms (Liu et al., 2024). In the quest for innovation, researchers are tirelessly developing highly efficient catalysts that promise to transform the hydrogen dissociation reaction, aiming for enhanced selectivity and cost-effectiveness to meet the demands of sustainable energy storage solutions.

Previously, Al_12_N_12_ nanocages were used to study as drug delivery of Favipiravir (an anti-COVID_19 drug) (Ibrahim et al., 2023). In selective detection of cadaverine, a biomarker for food freshness and environmental contamination, Cu-doped Al_12_N_12_ is used (de Sousa Sousa et al., 2024). Adsorption of gases is also studied by the help of Al_12_N_12_ nanocages (Nair et al., 2024). The adsorption of the Zolina drug on Al_12_N_12_ nanocages has also been investigated (Sheikhi et al., 2021). While these findings showcase the diverse applications of Al_12_N_12_, few studies have analyzed the single-atom catalytic activity of aluminum nitride nanocages. Al_12_N_12_ was selected because its curved, heteroatomic framework contains polar Al–N bonds and electronically distinct Lewis-acidic Al and Lewis-basic N sites, providing a fundamentally different metal-coordination environment from homonuclear carbon supports. The primary objective of this study is to design and investigate complexes of Al_12_N_12_ decorated with first-row transition metals, from Scandium to Zinc through Density Functional Theory (DFT), to explore the thermodynamic stability and electronic properties of these complexes. A key aspect of this research involves examining the types of interactions between the transition metals and the Al_12_N_12_ nanocages. Furthermore, it will be investigated how these metal-decorated Al_12_N_12_ nanocage fragments contribute to hydrogen dissociation, with the primary focus on setting low activation barriers for H_2_ molecule dissociation. The goal of this research is to better understand these processes to design transition metal-decorated Al_12_N_12_ nanocages for effective hydrogen dissociation. This marks an important step in enabling sustainable energy systems by facilitating the efficient dissociation of molecular hydrogen into reactive atomic species for downstream applications.

## 2 Computational details

In this study, all the calculations were performed on Gaussian 16 (Frisch et al., 2019), and for structural visualization, Gauss View 6.0 (Dennington et al., 2016) was used. Pure Al_12_N_12_ nanocage, transition metal-decorated Al_12_N_12_ complexes, and hydrogen-adsorbed TM@Al_12_N_12_ complexes have been optimized using the B3LYP-D3 functional and the 6-31+G (d,p) basis set. B3LYP functional serves as a hybrid approach in DFT, widely used for geometric and mechanistic investigations in computational studies of nanocages (Fallahi et al., 2018; Hussain et al., 2020) as well as other systems (Mahdy, 2015; Mohapatra et al., 2019; Zayed et al., 2019). Grimme’s Dispersion “D3” is also utilized in this study for the investigation of dispersion forces, which are critical for characterizing interactions (Grimme, 2006). Geometric optimization at the ground state (minima) is verified through frequency analysis, i.e., no imaginary frequencies are evident at stationary points on the potential energy surface (PES) in the reactants and products of H_2_TM@Al_12_N_12_ and TM@Al_12_N_12_ complexes. Imaginary or negative frequencies indicate transition states. We analyze the transition states using eigenvectors, which precisely characterize them.

To ensure the most stable conformations, the Al_12_N_12_ nanocage is subjected to exohedral substitution of first-row transition elements (from Sc to Zn), and the most stable configurations are selected for later computations. We evaluated various spin states to determine the most stable one. The values of spin-polarized calculations <S^2^> are computed using Equation 1 given below:

$$<S^{2}>=S\left(S+1\right)$$ (1)

here S = $\frac{1}{2}\times$ (half-filled electrons). The spin-polarized values vary with different spin states, which depend upon the number of unpaired electrons in the d-orbitals of transition metal atoms.

The TM@Al_12_N_12_ complexes in their most stable spin state were considered for further analysis of interaction energy and adsorption energy calculations. The interaction energy (E_int_) of TM@Al_12_N_12_ complexes is computed using Equation 2.

$$E_{\mathrm{int}\;}=E_{TM@{Al}_{12}N_{12}}-\left(E_{{Al}_{12}N_{12}}+{\;E}_{TM}\right)$$ (2)

here, ETM@Al_12_N_12._ means the energy of the TM@Al_12_N_12_ complex; the factor EAl_12_N_12_ is the energy of the bare Al_12_N_12_ nanocage, whereas ETM is the energy of the transition metal atoms.

The adsorption energy (E_ads_) for the H_2_ adsorption over TM@Al_12_N_12_ complexes is calculated using the Equation 3:

$$E_{ads}=E_{H_{2}TM@{Al}_{12}N_{12}}-\left(E_{TM@{Al}_{12}N_{12}}+E_{H_{2}}\right)$$ (3)

$EH_{2}TM@{Al}_{12}N_{12}$ is the total energy for adsorption of hydrogen over TM@Al_12_N_12._ *E*_*TM@*_ ${Al}_{12}N_{12}$ and $E_{H_{2}}$ denotes the energy of transition metal atoms decorated on Al_12_N_12_ and hydrogen, respectively.

The HOMO and LUMO energy gap ( $\left.H-L\right)$ is calculated by Equation 4:

$$H-L=E_{LUMO}-E_{HOMO}$$ (4)

here, $E_{LUMO}$ symbolizes the energy of the lowest unoccupied molecular orbital and $E_{HOMO}$ represents the energy of the highest occupied molecular orbitals. The ΔSCF approach is used to calculate the vertical ionization potential (VIP) and vertical electron affinity (VEA) values using Equations 5 and 6 respectively.

$$VIP=E\left(\text{cation}\right)-E\left(\text{neutral}\right)$$ (5)

$$EA=E\left(\text{neutral}\right)-E\left(\text{anion}\right)$$ (6)

FMO (Frontier molecular orbitals) analysis has been executed for the determination of perturbation in electronic levels, which represents a shift in the energy levels of molecular orbitals upon external influence, i.e., decoration/substitutin of TM atoms on the ${Al}_{12}N_{12}$ nanocage. NBO (natural bond orbitals) and VIP (Vertical ionization potential) have been computed at the B3LYP-D3/6-31+G (d,p) level of theory to ensure the charge transfer and chemical reactivity of TM@Al_12_N_12._ In order to reveal the nature of interactions among Transition metal atoms and the Al_12_N_12_ nanocage, IRI and QTAIM analyses have been performed using Multiwfn (Lu and Chen, 2012) in collaboration with VMD software (Humphrey et al., 1996). Density of states analysis was used to explore the electronic states available for electrons at each energy level. The DOS spectra comprising of TDOS and PDOS are obtained using PyMOlyze-1.1 software (O’boyle et al., 2008). EDD analysis performed using Multiwfn software provides deeper insights into the dissociation processes of H_2_ molecules by visualizing and quantifying the changes in electronic density. EDD for stable TM@ Al_12_N_12_ complexes is computed by subtracting the individual electron densities of H_2_ and TM@Al_12_N_12_ from the density of the combined H_2_TM@Al_12_N_12_ complexes, highlighting the charge redistribution effects.

The energy of reaction ΔE (amount of energy that changes during a reaction) and activation barrier E_a_ (minimum amount of energy required for a reactant to initiate the reaction) for hydrogen dissociation reaction is calculated using Equations 7 and 8 respectively.

$$\Delta E=E_{P}-E_{R}$$ (7)

$$E_{a}=E_{TS}\;–\;E_{R}$$ (8)

E_P_, E_R_ and E_TS_ represent the energy of products, reactants and transition state respectively.

## 3 Results and discussions

### 3.1 Geometrical parameters

The optimized Al_12_N_12_ nanocage has eight hexagonal and six tetragonal rings. The Al-N bond shared by two hexagonal rings has a bond length of 1.79 Å and the bond shared between a hexagonal ring and square ring has a bond length of 1.85 Å which is in accordance with the previous work on Al_12_N_12_ nanocage (He et al., 2022). Also, the bond length between two Aluminum atoms is 2.49 Å.

**FIGURE 1:**
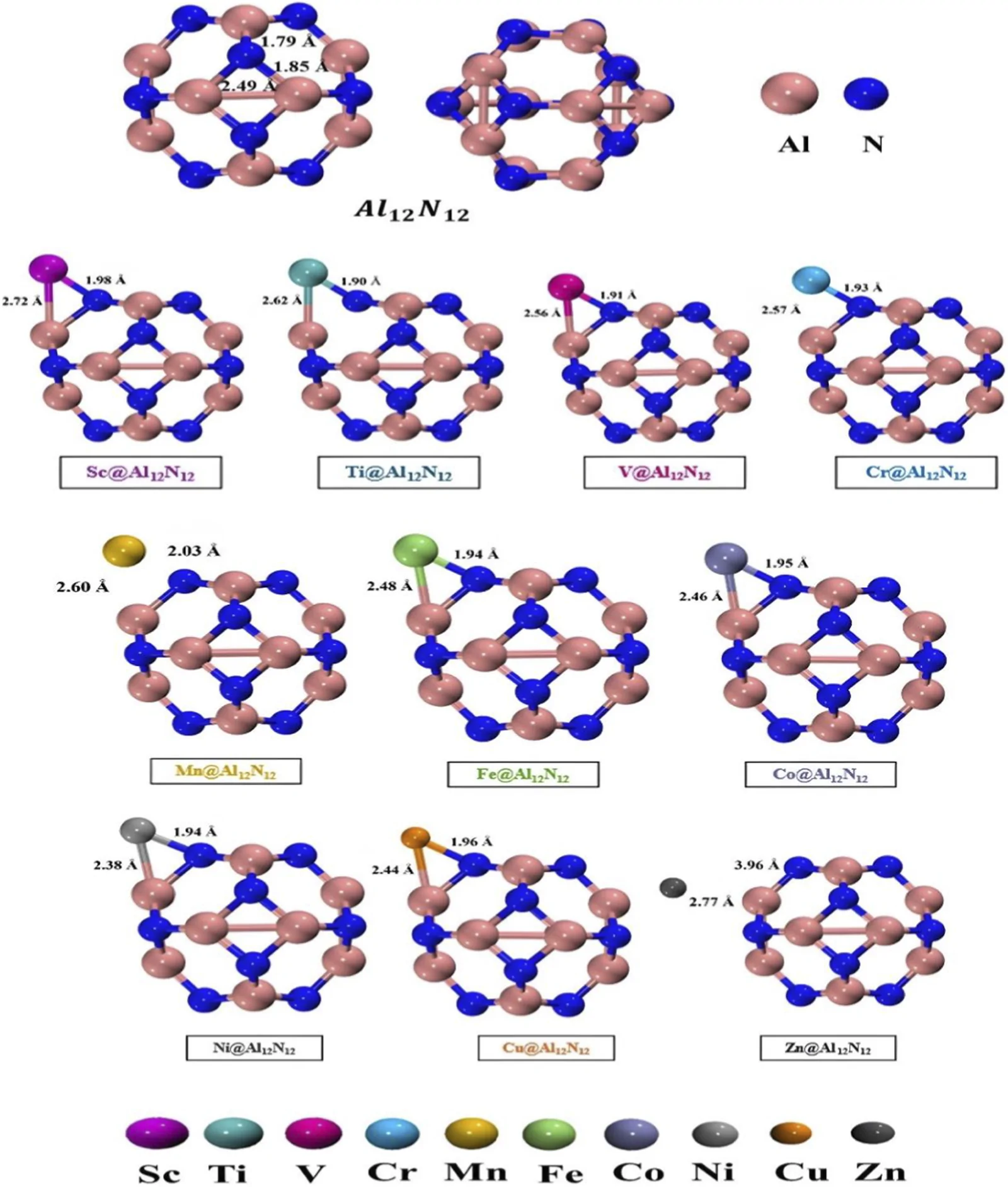
Visual representation of optimized geometries of pristine (pure) Al_12_N_12_ and transition metal decorated Al_12_N_12_.

First-row transition elements (Sc to Zn) are decorated on Al_12_N_12_ nanocage to get metal catalysts for studying H_2_ dissociation reaction. The geometric optimization and frequency calculations for decorated complexes of Al_12_N_12_ are performed at the same level of theory. Transition metal atoms may have multiple spin states because of partially filled d orbitals so spin polarized calculations are carried out for all the TM@Al_12_N_12_ complexes. The values of spin-polarized calculations <S^2^> (provided in Table 1) suggest that there is no spin contamination. The top and side views of optimized geometries of TM@Al_12_N_12_ is shown in Figure 1.

**TABLE 1:** Calculated structural parameters, bond lengths (X in units of Å), interaction energies (E_int_ in unit of eV), Gibbs free energies and spin-polarized values (<S^2^>) of TM decorated Al_12_N_12_ nanocage.

Complex	E_int_ (eV)	ΔG (eV)	X(Å) D_TM-Al_	X(Å) D_TM-N_	<S^2^>
Sc@Al_12_N_12_	−2.37	−2.05	2.72	1.98	0.75
Ti@Al_12_N_12_	−2.56	−2.04	2.62	1.90	2.00
V@Al_12_N_12_	−2.31	−1.99	2.56	1.91	3.78
Cr@Al_12_N_12_	−2.41	−2.08	2.57	1.93	6.10
Mn@Al_12_N_12_	−1.23	−0.90	2.60	2.03	8.78
Fe@Al_12_N_12_	−2.07	−1.73	2.48	1.94	6.02
Co@Al_12_N_12_	−1.69	−1.37	2.46	1.95	3.76
Ni@Al_12_N_12_	−1.63	−1.30	2.38	1.94	2.00
Cu@Al_12_N_12_	−1.29	−0.96	2.44	1.96	0.75
Zn@Al_12_N_12_	−0.38	−0.13	2.77	3.96	0

The TM atoms are exohedrally decorated on the top of the Al_12_N_12_ nanocage. Different positions have been explored for exohedral substitution, but the ‘b66’ position was found to be the most stable for doping. Apart from Mn and Zn, the metal atoms are chemically bonded with both Al and N atoms of the Al_12_N_12_ nanocage. No chemical link is observed between Mn/Zn and the Al_12_N_12_ nanocage, which demonstrates weaker interactions between them. Also, in the case of Cr@Al_12_N_12_, the chromium atom is bonded only to the nitrogen atom.

The transition metal atoms in the middle of the series exhibit higher spin multiplicities than those of the other transition metals. It is perceived that most spin states undergo a monotonic increase from doublet (for Sc@Al_12_N_12_) to sextet (for Mn@Al_12_N_12_) and then are followed by a decrease from quintet (for Fe@Al_12_N_12_) to singlet (for Zn@Al_12_N_12_). The results are consistent with earlier findings by Arshad et al. (Arshad et al., 2018).

The bond lengths in TM-Al and TM-N are considered, as transition metal atoms are decorated on a bond between Al and N. In Sc@Al_12_N_12_, the bond length between Sc and Al is 2.72 Å and, similarly, between Sc and N is 1.98 Å, as tabulated in Table 1. These values of bond lengths confirm the presence of covalent bonds between Sc and Al/N atoms. The interaction distance decreases as the atomic number of TM metals increases from left to right. This trend is disrupted by Mn- and Zn-decorated complexes. A higher interaction distance is observed for Mn and Zn@Al_12_N_12_ complexes because of weaker interaction forces in Mn/Zn and Al_12_N_12_ nanocage.

### 3.2 Thermodynamic stability

To evaluate the stability of the resulting complexes, Equation 1 is used to compute the interaction energies. The negative interaction energy values (see Table 1) suggest that doping of transition metals over Al_12_N_12_ nanocage is energetically feasible and the resultant compounds are thermodynamically stable. The E_int_ values range from −2.56 eV (for Ti@Al_12_N_12_) to −0.38 eV (for Zn@Al_12_N_12_). The lowest interaction energy value and increased interaction distance for Zn suggest weak van der Waals interactions. The interaction energies are also calculated using a mixed-basis approach in which def2-SVP is applied to the transition-metal atom, while 6-31+G(d,p) was retained for the Al_12_N_12_ nanocage. Thus, except for Zn, all the transition metals from Sc to Cu exhibit negative interaction energies as shown in Supplementary Table S1 with mixed/split basis sets, confirming that their doping on Al_12_N_12_ is energetically favorable and that the principal screening conclusion is not an artifact of the original basis set.

Higher values of interaction energy enhance hydrogen adsorption on the catalyst (TM@Al_12_N_12_), thereby increasing catalytic activity. Higher interaction energy is recommended in single-atom catalysis because it creates a robust chemical interaction between metal atoms and nanocage, maintaining stability and reducing aggregation/clustering.

In order to distinguish electronic binding strength from thermodynamic favorability, Gibbs free energies are calculated for the formation of the TM@Al_12_N_12_ complexes. Unlike electronic interaction energy, ΔG incorporates zero-point vibrational energy, finite-temperature thermal corrections and entropy. All ten complexes exhibit negative Gibbs binding free energies, with values ranging from −2.08 eV for Cr@Al_12_N_12_ to −0.13 eV for TM@Al_12_N_12_. The strongly negative values obtained for Cr (−2.08 eV), Sc (−2.05 eV), Ti (−2.04 eV) and V (−1.99 eV) indicate a highly favorable metal-cage association as shown in Table 1. Fe, Co, and Ni decoration also remains favorable, with values of −1.73, −1.36, and −1.30 eV, respectively, while Mn and Cu exhibit moderately favorable values of −0.90 and −0.96 eV, respectively. By contrast, the small negative value obtained for Zn@Al_12_N_12_ (−0.13 eV) indicates only marginal thermodynamic favorability and should be interpreted cautiously. Overall, the Gibbs free-energy results demonstrate that formation of the investigated TM@Al_12_N_12_ complexes is thermodynamically favorable under the modeled gas-phase standard conditions, while the magnitude of the driving force is strongly dependent on the electronic configuration of the supported metal.

This bonding strength can also be quantitatively analyzed using an energy threshold of −0.5 eV (Kream Alaarage et al., 2023). Interaction energies above −0.5 eV indicate strong bonding, while values below −0.5 eV reflect weaker interactions, consistent with the stability required for catalytic applications (Farrokhpour et al., 2019). Table 1 shows the interaction energy values. To evaluate catalyst stability beyond static interaction energies, we performed Gibbs free-energy calculations, which show that the metals are not aggregated due to more negative values. and AIMD simulations for the two leading candidates, Sc@Al_12_N_12_ and Ti@Al_12_N_12_ that shows the kinetic stability and are given in Supplementary Figure S5. The calculated Gibbs free energies of metal anchoring were negative, indicating that forming both decorated structures remains thermodynamically favourable after including thermal corrections. During the approximately 5.2 ps AIMD simulations, the total energies exhibited bounded fluctuations without systematic drift. The Al_12_N_12_ cages maintained their structural integrity, while the Sc and Ti atoms remained associated with their anchoring regions without observable desorption or pronounced migration. These observations support the short-timescale thermal stability of the isolated metal sites. Due to a shortage of resources, we were able to perform AIMD for two catalysts.

### 3.3 NBO analysis

NBO analysis offers an effective approach for evaluating both intramolecular and intermolecular bonding, while also serving as a reliable tool for investigating charge transfer and interactions within molecular systems (Sakr et al., 2022). The electropositive nature of transition metals and their charge transfer to the Al_12_N_12_ nanocage are expected to induce polarization in the TM atoms. TMs have been shown to have a positive charge, indicating that they give the Al_12_N_12_ nanocage excess electrons. The charge transfer amounts for Sc@Al_12_N_12_, Ti@Al_12_N_12_, V@Al_12_N_12_, Cr@Al_12_N_12_, Mn@Al_12_N_12,_ Fe@Al_12_N_12,_ Co@Al_12_N_12_, Ni@Al_12_N_12_, Cu@Al_12_N_12_ and Zn@Al_12_N_12_ are 0.58, 0.51, 0.48, 0.47,0.46,0.39, 0.35, 0.33, 0.31, 0.24 |e| respectively.

As we move from scandium to Zinc, atomic size decreases, leading to a stronger nuclear charge that holds electrons closer to the nucleus. Therefore, scandium exhibits a higher charge-transfer value than other transition metals. Strong nuclear charge reduced shielding effect, and atomic size collectively result in a decrease in charge transfer. Cobalt (Co) exhibits less charge transfer because of its high ionization potential; therefore, high energy is required to remove a valence electron. On the other hand, Ni and Cu are less energetically favorable donors of electrons due to their filled d orbitals. Zn has transferred the least charge to the cage because of its smaller size. The charge-transfer values are shown in Table 2. The calculated charge transfer from the transition-metal centers to the Al_12_N_12_ cage indicates that the nanocage functions as an electronically active support rather than an inert anchoring substrate. This interpretation is consistent with the broader observation that the local coordination environment and neighboring-site density can substantially modify the electronic structure and adsorption behavior of atomically dispersed metal centers (Liao et al., 2025). Strong intercomponent coupling at heterogeneous interfaces has likewise been reported to redistribute electron density and optimize intermediate binding (Fan et al., 2026). In the present systems, such support-induced electronic modulation explains the metal-dependent variation in H_2_ adsorption, H–H bond activation and stabilization of the resulting hydrogen atoms.

**TABLE 2:** Calculated values of NBO charges (Q in units of |e|), E_HOMO_, E_LUMO_, energy gaps (H-L), Vertical ionization potential (IP), Vertical electron affinity (EA), Hardness (η) and Softness (s) of pristine and TM-decorated Al_12_N_12_ complexes.

Complex	Q |e|	E_HOMO_ (eV)	E_LUMO_ (eV)	H-L (eV)	VIP (eV)	VEA (eV)	η (eV)	S (eV)
Al_12_N_12_	​	−6.50	−2.64	3.86	--	--	1.93	0.26
Sc@Al_12_N_12_	0.58	−4.12	−2.40	1.72	5.63	1.36	0.86	0.58
Ti@Al_12_N_12_	0.51	−4.65	−2.42	2.23	5.85	1.36	1.11	0.45
V@Al_12_N_12_	0.48	−5.17	−2.43	2.74	6.32	1.35	1.37	0.37
Cr@Al_12_N_12_	0.47	−5.58	−2.49	3.09	7.88	1.39	1.54	0.32
Mn@Al_12_N_12_	0.46	−4.67	−2.61	2.07	6.22	1.52	1.03	0.48
Fe@Al_12_N_12_	0.39	−4.62	−2.59	2.03	7.41	1.51	1.01	049
Co@Al_12_N_12_	0.35	−4.59	−2.58	2.01	6.13	1.51	1.01	0.50
Ni@Al_12_N_12_	0.33	−4.65	−2.56	2.09	6.24	1.47	1.04	0.48
Cu@Al_12_N_12_	0.31	−4.57	−2.55	2.02	6.17	1.46	1.01	0.50
Zn@Al_12_N_12_	0.24	−6.37	−2.61	2.07	7.63	1.48	1.90	0.26

### 3.4 Frontier molecular orbital (FMO) analysis

**FIGURE 2:**
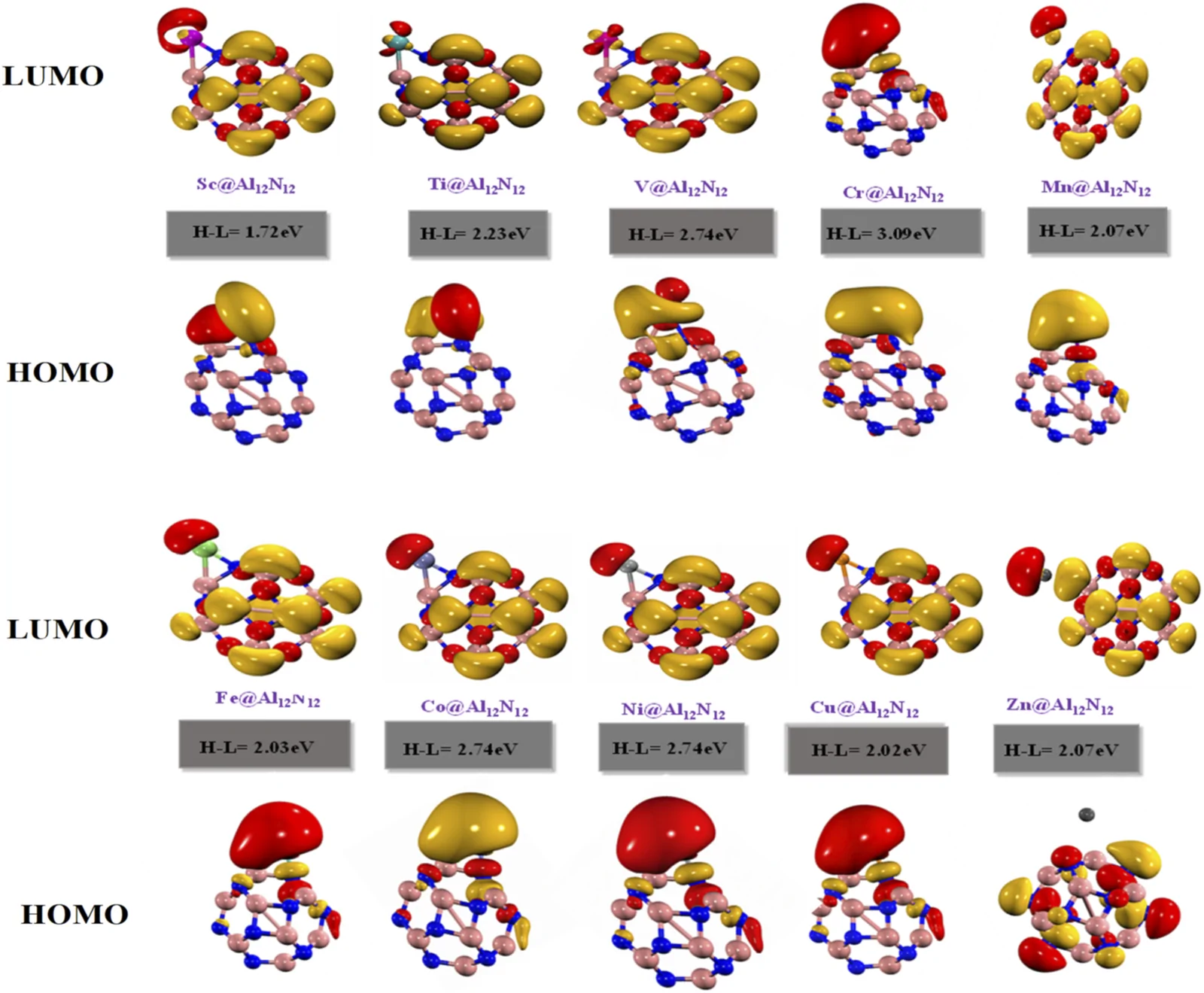
Frontier molecular orbitals (FMOs) analysis for transition metal decorated Al_12_N_12_ nanocage depicting the HOMO and LUMO energy levels and their resulting energy band gap (H–L) values.

The prediction of electronic spectra, chemical and kinetic stability, and electrical and optical properties of molecules strongly depends on frontier molecular orbitals (FMOs). Analyzing a molecule’s FMOs, specifically the highest occupied molecular orbital (HOMO) and the lowest unoccupied molecular orbital (LUMO), provides valuable insights into its electronic properties and chemical reactivity (Jumabaev et al., 2024). Figure 2 illustrates FMO analysis of all complexes.

The doping of transition elements onto the Al_12_N_12_ nanocage has resulted in a reduction in the H-L gap as compared to the pure Al_12_N_12_nanocage (3.86 eV). Excess electrons introduced by transition elements cause the H-L gap to get smaller. It results in new HOMOs being created. Compared to pure Al_12_N_12_ nanocage, TM@Al_12_N_12_ complexes exhibit improved electrical properties, as evidenced by their reduced H-L gap. The HOMO (LUMO) energies for Sc@Al_12_N_12_, Ti@Al_12_N_12_, V@Al_12_N_12_, Cr@Al_12_N_12_, Mn@Al_12_N_12_, Fe@Al_12_N_12_,Co@Al_12_N_12_, Ni@Al_12_N_12_, Cu@Al_12_N_12_, Zn@Al_12_N_12_ are −4.12 (−2.40), −4.65(-2.42), −5.17 (−2.43), −5.58 (−2.49),-4.67(-2.61), −4.59 (−2.58), −4.59 (−2.56), −4.57 (−2.55) and −6.37 (-2.61) | eV |, respectively. Notably Sc@Al_12_N_12_exhibits the lowest H-L gap value (1.72 eV), while Cr@Al_12_N_12_ exhibits the largest H-L gap value (3.09 eV) as mentioned in Table 2. The graphical representation of HOMO-LUMO isodensities is given in Figure 2.

The increment in HOMO energy alongside the drop in LUMO energy results in a decrease in H-L gap values. The energy values of the HOMO increase because of the presence of diffused excess electrons, which consequently lowers H-L gap values. These new energy levels in TM@Al_12_N_12_ are referred to as new HOMOs relative to those of the undecorated Al_12_N_12_. These alterations significantly change the Al_12_N_12_ nanocage’s electrical properties. Furthermore, the charge transfer between the Al_12_N_12_ cage and TMs is facilitated by the drop in H-L values inducing a significant interaction between Al_12_N_12_ and TMs by making it energetically easier for electrons to move between orbitals.

First, an increase in ionization potential and a decrease in transition-metal (TM) atomic radius contribute to increased complex stability, leading to a larger HOMO–LUMO (H–L) gap from Sc to Zn. Ionization potential-the energy required to remove an electron from the valence shell-helps explain why Sc@Al_12_N_12_ exhibits the lowest H–L gap, whereas Zn@Al_12_N_12_, with the highest ionization potential, shows the largest H–L gap. The highest IP was observed for Zn@Al_12_N_12_, indicating that this complex is highly stable and that removing a valence electron requires a significant amount of energy. Conversely, Sc@Al_12_N_12_ has the lowest IP, correlating with its lowest H-L gap. Finally, HOMO electron densities are clearly localized on the TMs, whereas LUMO electron densities are concentrated on the cage framework.

### 3.5 Global energy descriptors

The doping response of isomers is often assessed through global reactivity parameters, as these significantly influence their frontier molecular orbitals (Ramírez-Martínez et al., 2023). The LUMO and HOMO energy values determine the electron affinity (EA) and ionization potential (IP), which are analogous to the electron-accepting and electron-donating properties of molecules, respectively (Pandey et al., 2021).

The vertical IP and EA computed through the ΔSCF approach for the designed M@Al_12_N_12_ complexes disclose a clear dependence on the individuality of the doped transition metal. The IP values vary significantly across the series, from 5.63 eV for Sc@Al_22_N_12_ to 7.88 eV for Cr@Al_12_N_12_. The complexes Zn@Al_22_N_12_ (7.63 eV) and Fe@Al_22_N_12_ (7.41 eV) are especially notable as being quite hard to ionize. This behavior is not monotonic throughout the series; rather, it increases toward chromium, dips around manganese and cobalt, and then rises again toward zinc. This trend can be explained by the relative stability of the half-filled (Cr) and fully filled (Zn) 3 days subshells, both of which are known to be more resistant to additional electron removal than other members of the same group. On the other hand, regardless of the metal doped, the vertical EA is nearly constant across the series, confined to a narrow range of 1.35–1.52 eV. When combined, these findings show that the choice of dopant metal has a significant impact on how easily the complex can be oxidized, with Sc-doped and Ti-doped systems emerging as the most readily ionized and Cr-doped and Zn-doped systems as the most resistant.

The band gap correlates with the molecule’s softness and hardness, which provides insight into its reactivity. The molecule’s hardness is directly correlated with the band gap and inversely correlated with its reactivity. The band gap increases with increasing hardness, resulting in reduced reactivity and lower charge transfer. A molecules’ softness and polarizability increase with a smaller band gap, thereby increasing the molecules’ reactivity (Maqsood et al., 2022).

It is obvious from the results that transition metals decrease the hardness of Al_12_N_12_ nanocage while increasing its softness. Transition metals increase softness values by up to 0.58 eV. On the other hand, hardness decreases by up to 0.86 eV. Furthermore, Zn@Al_12_N_12_ shows the highest hardness value of 1.90 eV while the lowest softness value of 0.26 eV. Due to the small size of zinc, there is great difficulty in charge transfer between zinc atom and cage resulting in the lesser softness and higher hardness values. All global energy parameters are displayed in Table 2.

### 3.6 Density of states (DOS) analysis

**FIGURE 3:**
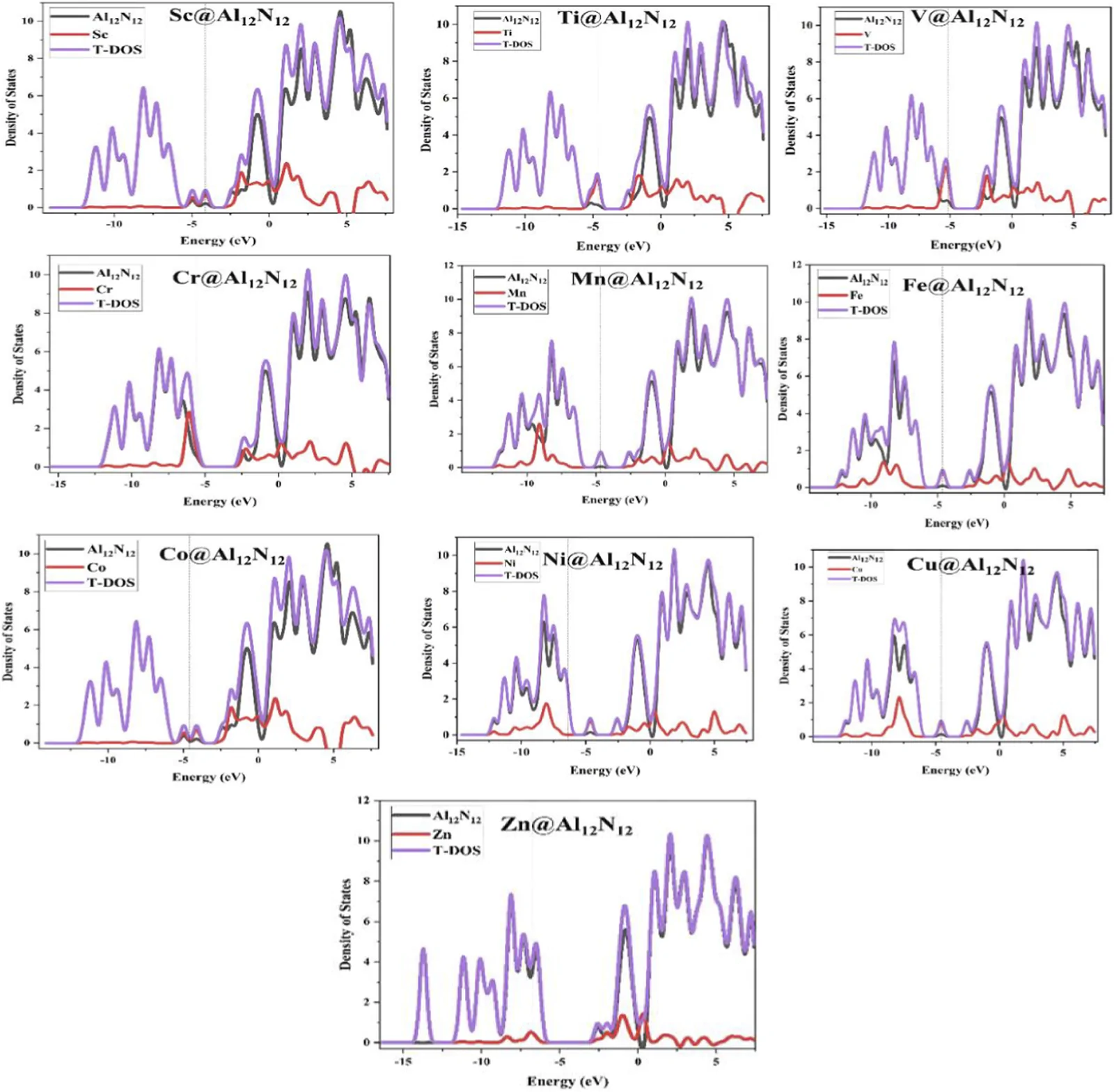
Density of states (DOS) spectra for the pristine and TM@Al_12_N_12_ complexes.

The density of states (DOS) characterizes the energy levels per unit energy increment and their energy composition. The corresponding plots provide a comprehensive description of the whole orbitals system (Fradi et al., 2021). In the given spectra, black lines indicate the contribution of the pristine nanocage, while the red lines exhibit contribution of the TMs. These black and red lines are known as PDOS (Partial Density of States) curves, while the purple line is TDOS (Total Density of States) curve. It is clearly evident from all the spectra that doping of TMs is an effective strategy to reduce H-L gap and create new, higher-energy HOMOs. The transfer of charge from the TMs to the Al_12_N_12_ nanocage during the formation of new HOMOs is confirmed by the PDOS spectra. The positions of these newly formed HOMOs are indicated by the vertical dashed lines in the DOS spectra. The contribution of TMs to the drop in the H-L gap and the subsequent change in the electronic properties of Al_12_N_12_ nanocage are clearly demonstrated by the DOS spectra. These observations lead to the fact that doping of TMs has a major role in the alteration of electronic properties of Al_12_N_12_ nanocage. DOS spectra of all complexes are shown in Figure 3.

### 3.7 IRI analysis

The ability of IRI analysis to clearly show the smooth transitions between weak interaction and chemical bonds makes it useful for studying chemical reactions (Sagaama et al., 2021). Because interatomic interactions occur often in molecular systems, IRI is highly helpful in the study of numerous molecular structures since it can identify a various molecular interaction zone. Depending on the electron density, IRI is useful for detecting chemical bonds in molecules (Sravanthi et al., 2023). The IRI analysis workflow consist of 3D-isosurfaces and 2D plots. The 3D-isosurfaces has multiple color codes which describe the interaction among the atoms in the complexes. For instance, the green color in the iso-surfaces display a zone of weak interaction, the blue color code in the iso-surfaces indicates a zone of high electron density that strengthens the bond, and the color red in the iso-surfaces show the steric effect or repulsion and can be calculated using Equation 9:

$$\text{IRI}\left(r\right)=\frac{\left|\nabla \rho \left(r\right)\right|}{{\left[\rho \left(r\right)\right]}^{a}}$$ (9)

In the above equation, the factor *ρ* is electron density and **r** is coordinate vector. This study uses the (sinλ2)ρ function to identify different interaction zones, where Steric repulsive forces are expressed as (sinλ2)ρ 0.

The blue-colored covalent bond regions in the IRI iso-surface between the aluminum atoms of the Al_12_N_12_ nanocage and the transition metal atoms (Sc to Cu) indicate a high electron density and interaction strength.

**FIGURE 4:**
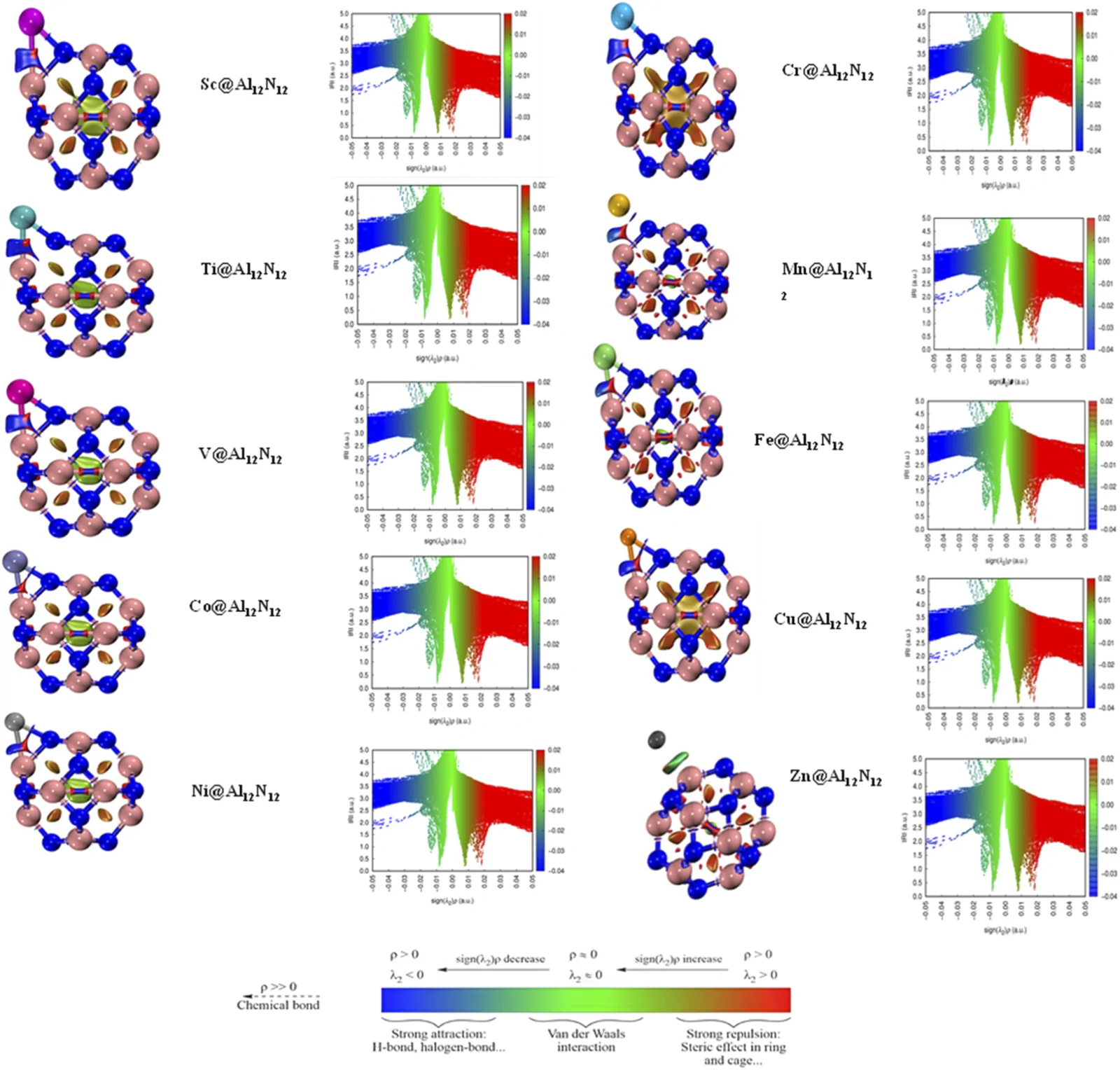
3D iso surfaces and 2D-IRI graphs of TM@Al_12_N_12_ complexes.

Additionally, the chemisorption of transition metal atoms onto the Al_12_N_12_ nanocage surface is confirmed by the existence of covalent interactions. However, there is a green patch between zinc and aluminum, indicating that there are weak van der Waals forces between them. All isosurfaces and graphs are illustrated in Figure 4.

### 3.8 QTAIM analysis

To obtain an extensive, in-depth understanding of molecular interactions, a detailed Quantum Theory of Atoms in Molecules (QTAIM) analysis was carried out on each complex. These analyses were performed on the TM@Al_12_N_12_ complexes. The color codes in the IRI analyses indicate that the TMs are making interactions with atoms or multiple atoms of the Al_12_N_12_ nanocage, and the precise nature of these interactions is confirmed by using QTAIM analysis. The orange spots located between TMs and Al_12_N_12_ represent the bond critical (BC) point.

**FIGURE 5:**
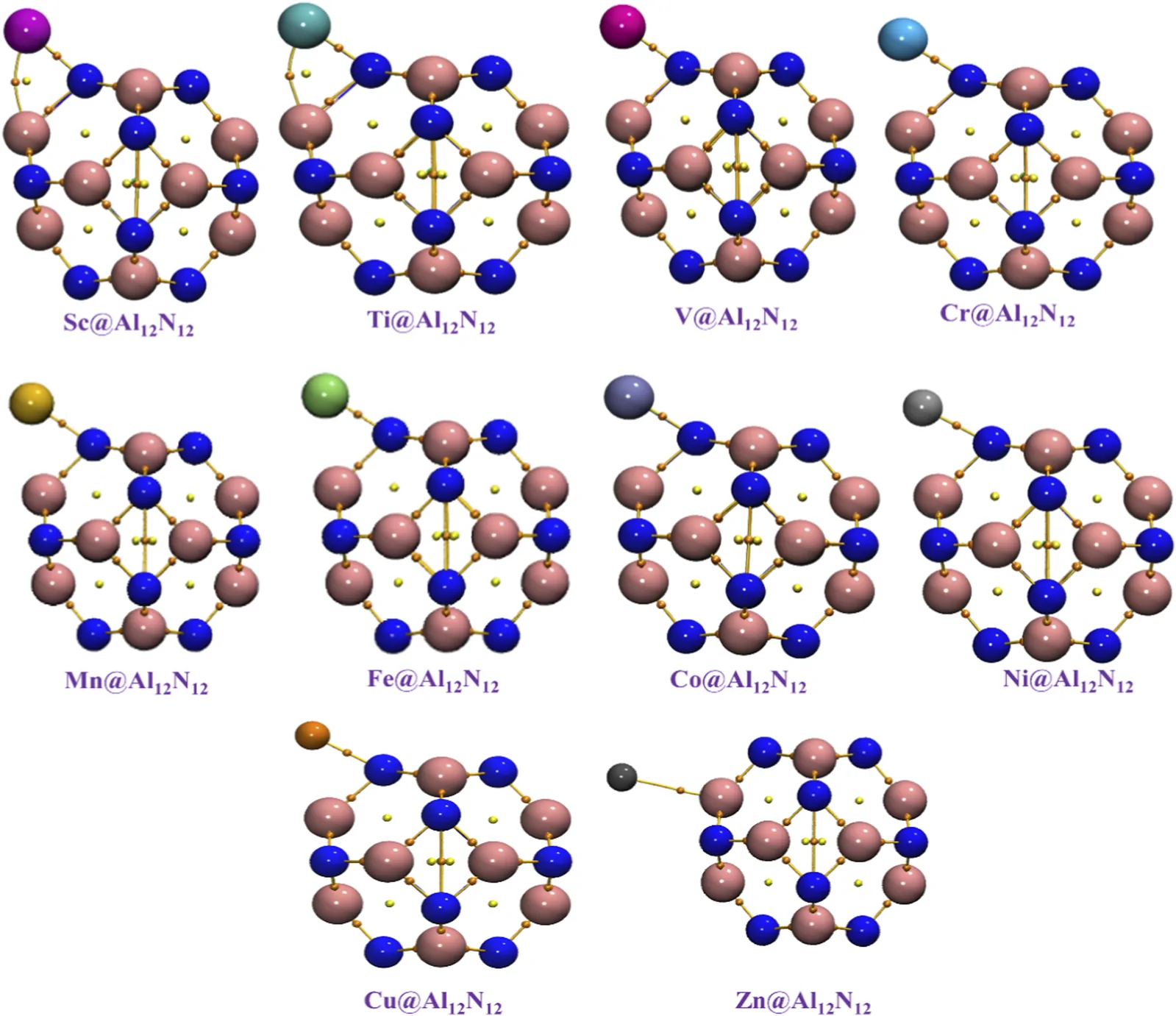
QTAIM topological analysis of TM@Al_12_N_12_ complexes displaying the BCPs.

Figure 5 shows the molecular graph of these structures. Assessing and ensuring the strength of intermolecular interactions depends on this BC point.

The appearance of BC-points in a molecule indicates that chemical bonds have formed between the cage and the TMs, which also indicates the mutual transfer of electron density (Ahmed et al., 2024). The following formula given in Equations 10 and 11 can be used to express the values of the BC-points:

$$H\left(r\right)=G\left(r\right)+V\left(r\right)$$ (10)

$$\left(\frac{1}{4}\;\right)\nabla^{2}p_{r=}2\;G{\left(r\right)}_{r}+V\left(r\right)$$ (11)

The kinetic energy density G(r), potential energy density V(r), interaction energy E_interactions_, electron density ρ(r), electron density Laplacian ∇2ρ(r), and the ratio |V|/G can all be used to describe the nature of interactions. Robust covalent bonds are indicated by negative values of ∇^2^p, a ratio of–G (r)/V (r)less than 1, H(r) less than 0 au, and p more than 0.1au. Conversely, positive values of ∇^2^p(r), a ratio of–G (r)/V (r)more than1, H(r) greater than 0 au, and p(r) smaller than 0.1 au are characteristic of van der Waals interactions. Complexes with H(r) less than 0 au and positive values for the Laplacian of electron density ∇^2^p(r) exhibit both covalent and electrostatic interactions (Yaqoob et al., 2024).

The electron density values for the TM@Al_12_N_12_ complexes range from 0.0207 to 0.1305 au. Among them, Ti@ Al_12_N_12_ has the greatest p value indicating covalent interactions, demonstrating the strongest interactions while Zn@Al_12_N_12_ has lowest p value indicating the weakest interaction. The ratio of–G(r)/V (r) values lie between 0.4721 and 0.8779 for all complexes. Ti@Al_12_N_12_ has lowest–G(r)/V (r) value, indicating a strong interaction between Ti atom and Al_12_N_12_ nanocage. Moreover, its negative ∇^2^p(r) confirms a strong interaction of titanium with Al_12_N_12_ nanocage.

The QTAIM analysis’s results are consistent with the thermodynamic data of TM@Al_12_N_12_ complexes, which show that the Ti@Al_12_N_12_ complexes have the strongest doping when compared to other TM@Al_12_N_12_ complexes due to its highest negative E_int_ values. The values of QTAIM analysis of all complexes are shown in Table 3.

**TABLE 3:** QTAIM (Quantum theory of atoms in molecules) topological parameters evaluated at the Bond critical points (BCPs) to characterize bond interactions in all designed TM@Al_12_N_12_ complexes.

Complexes	*ρ*(r)	∇^2^ *ρ*(r)	G(r)	K(r)	V(r)	H(r)	-G(r)/V(r)
Sc@Al_12_N_12_	0.0370	−0.0021	0.0088	0.0093	−0.0181	−0.0093	0.4854
​	0.1124	0.3235	0.1141	0.0332	−0.1473	−0.0332	0.7745
Ti@Al_12_N_12_	0.0394	−0.0051	0.0108	0.0121	−0.0228	−0.0121	0.4721
​	0.1305	0.4002	0.1428	0.0428	−0.1856	−0.0428	0.7695
V@Al_12_N_12_	0.1201	0.4367	0.1379	0.0287	−0.1667	−0.0287	0.8276
Cr@Al_12_N_12_	0.1112	0.4609	0.1355	0.0203	−0.1558	−0.0203	0.8697
Mn@Al_12_N_12_	0.0887	0.3575	0.1038	0.0144	−0.1182	−0.0144	0.8779
Fe@Al_12_N_12_	0.1084	0.3996	0.1273	0.0274	−0.1547	−0.0274	0.8228
Co@ Al_12_N_12_	0.1043	0.3641	0.1236	0.0326	−0.1561	0.0326	0.7915
Ni@ Al_12_N_12_	0.1056	0.3225	0.1237	0.0431	−0.1668	−0.0431	0.7416
Cu@ Al_12_N_12_	0.0974	0.2869	0.1162	0.0445	−0.1608	−0.0445	0.7231
Zn@Al_12_N_12_	0.0207	0.0033	0.0059	0.0051	−0.0110	−0.0051	0.5371

### 3.9 Hydrogen molecule adsorption over TM@Al_12_N_12_ complexes

The H_2_-adsorption over TM-atom catalysts is carried out in vertical as well as horizontal orientation. Mn and Zn@Al_12_N_12_ complexes are not used for hydrogen adsorption due to lack of chemical interaction and lowest interaction energy between Mn/Zn atoms and nanocage. The interaction energy is −1.23 and −0.38 eV for Mn and Zn respectively. The inherent stability of Mn (d^5^) and Zn (d^10^), due to their half-filled and fully filled d-orbitals respectively, limits charge transfer between the metal atoms and the Al_12_N_12_ nanocage, leading to weaker interaction energies. So, Mn/Zn decorated complexes are not suitable for efficient adsorption of H_2_ over TM@Al_12_N_12._

**FIGURE 6:**
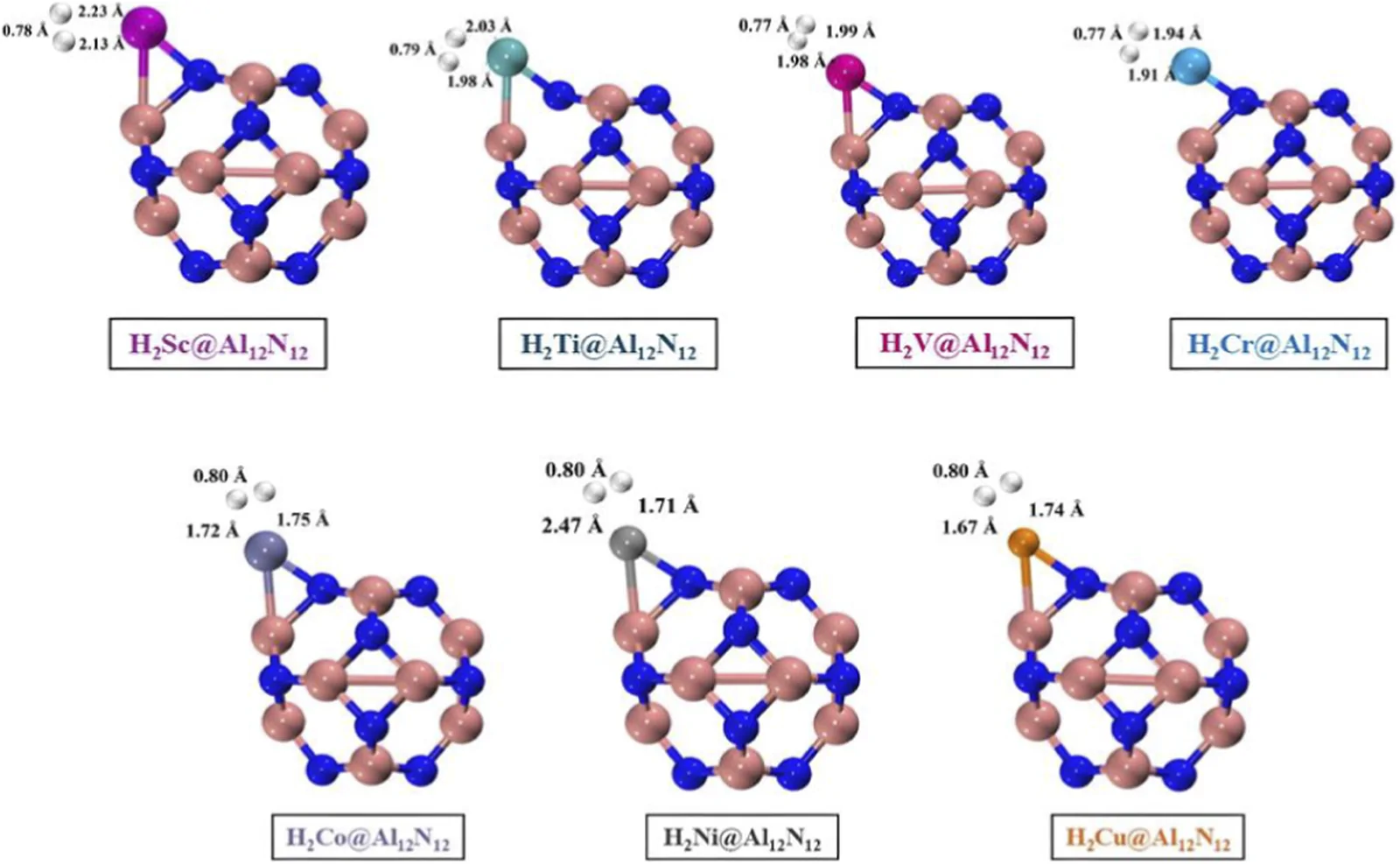
Optimized structures of stable hydrogen molecule-adsorbed TM@Al_12_N_12_ complexes.

The distance between two hydrogen atoms in a hydrogen molecule adsorbed over TM@Al_12_N_12_ complexes ranges from 0.77 to 0.80 Å. In all the H_2_-TM@Al_12_N_12_ complexes, the H and H bond distance is greater than isolated Hydrogen molecule (bond length of H-H: 0.74 Å) which is due to stronger interaction between TM-atoms and H-atoms that eventually weakens the H-H bond and increases its bond length as shown in Figure 6.

The increase in bond length causes H_2_ activation occurring at individual metal atom sites. Various spin state computations are executed for H_2_-TM@Al_12_N_12_ complexes, and further calculations are carried out on the stable spin state. The adsorption energy E_ads_refers to the amount of energy released by hydrogen atom to get adsorbed on the TM@Al_12_N_12_. Negative values of E_ads_ signify an exothermic reaction, indicating that hydrogen adsorption is thermodynamically favorable process (Sarfaraz et al., 2023). The E_ads_ values of H_2_-TM@Al_12_N_12_ complexes are computed using Equation 3 and summarized in Table 4. H_2_ScTM@Al_12_N_12_ complex has the lowest E_ads_ value (−0.08 eV) while H_2_Cr@Al_12_N_12_ has the highest adsorption energy value (−0.30 eV). In the case of Fe@Al_12_N_12_, hydrogen adsorption is unfavorable due to positive adsorption energies; thus, it was not selected for subsequent calculations.

**TABLE 4:** Calculated Adsorption energies (E_ads_), Activation barrier (E_a_), and Energy of reaction (ΔE) in electron volts (eV) for the adsorption and reaction pathways of all H_2_TM@Al_12_N_12_ complexes.

Complexes	E_ads_ (eV)	E_a_ (eV)	ΔE (eV)
H_2_Sc@Al_12_N_12_	−0.08	0.010	−1.64
H_2_Ti@Al_12_N_12_	−0.23	0.005	−1.38
H_2_V@Al_12_N_12_	−0.29	0.033	−1.34
H_2_Cr@Al_12_N_12_	−0.31	0.224	−0.82
H_2_Co@Al_12_N_12_	−0.10	0.356	−1.14
H_2_Ni@Al_12_N_12_	−0.13	0.330	−0.91
H_2_Cu@Al_12_N_12_	−0.16	0.396	−0.32

### 3.10 Dissociation of hydrogen over TM@Al_12_N_12_ catalyst

The dissociative adsorption of hydrogen molecules is a key reaction on catalytic surfaces, as it involves both the cleavage of a covalent bond and the concurrent formation of new atomic bonds. A potential field called the energy barrier (E_a_), sometimes referred to as activation energy, may serve as a crucial criterion for regulating or localizing the transference of charged particles (electrons) (Lei et al., 2017). Thus, it is vital to overcome the E_a_barrier for transfer of charge.

The Ti@Al_12_N_12_ complex has the lowest E_a_ value among all the TM@Al_12_N_12_ complexes, at 0.005 eV, while the Cu@Al_12_N_12_ complex has the maximum E_a_ value, at 0.396 eV. The energy barrier values for Sc@Al_12_N_12_, V@Al_12_N_12_, Cr@Al_12_N_12_, Co@Al_12_N_12_, and Ni@Al_12_N_12_ are 0.10, 0.033, 0.224, 0.356, and 0.33 (eV), respectively.

Ti@Al_12_N_12_ showed the highest catalytic activity, with the lowest activation barrier of 0.005 eV, after evaluating the effectiveness of different catalysts in promoting the hydrogen dissociation process (HDR). The process is considered effectively barrierless within the accuracy of the computational method. Comparable nonactivated H_2_ cleavage has been reported for several metal-based catalysts (Table 5). Thus, H–H cleavage is unlikely to be rate determining, although overall catalytic performance will also depend on H_2_ accessibility, hydrogen transfer, product desorption, catalyst regeneration, and active-site stability. Ti-metal having Ti-Al and Ti-N bond lengths of 2.62 Å and 1.90 Å, respectively, is initially adsorbed on the Al_12_N_12_ nanocage at b66 position, with an interaction energy of −2.56 eV. For the exploration of H_2_-molecule dissociations, it is adsorbed horizontally over the Ti@Al_12_N_12_ catalyst. The thermodynamic stability of the H_2_Ti@Al_12_N_12_ complex is evident by the adsorption energy of −0.23 eV.

**TABLE 5:** Comparison of the calculated H_2_-dissociation activation barrier over Ti@Al_12_N_12_ with those reported for representative catalysts in the literature.

Study and reference	Catalyst system	Reported H_2_-dissociation barrier	Evidence and interpretation
Present work	Ti@Al_12_N_12_	0.005 eV (0.48 kJ mol^-1^)	DFT transition-state and IRC analysis. The value is effectively barrierless within computational accuracy
Alonso et al. (2021)	Ru_1_@S-1, Rh_1_@S-1 and Pt_1_@S-1	Barrierless (ΔE‡ = 0 eV)	Periodic DFT screening of zeolite-confined single atoms; spontaneous or barrierless H_2_ cleavage was reported for Ru, Rh and Pt sites
Rodríguez-Kessler et al. (2018)	Pd_8_V, Pd_10_V and Pd_11_V clusters	Barrierless	Cluster DFT calculations identified specific V-doped Pd sizes capable of barrierless H_2_ dissociation
Zhu et al. (2021)	Ni_8_/In_2_O_3_ and Ni_1_/In_2_O_3_	Ni_8_: submerged/barrierless relative to gas-phase H_2_ Ni_1_: ≈0.11 eV	Experimental CO_2_-hydrogenation catalyst characterized by H_2_-TPR, XPS, XAS and EPR, supported by DFT. The reference state is separated H_2_ plus catalyst
Lushchikova et al. (2021)	Cu_5_^+^ cluster	TS at −0.14 or −0.08 eV relative to separated reactants	IRMPD spectroscopy with H_2_/D_2_ and DFT. Dissociated hydrogen dominates because the cleavage transition states are submerged below the reactant asymptote
Busnengo and Martínez (2008)	W(100) and W(110)	Nonactivated	Six-dimensional potential-energy surfaces and trajectory calculations reproduced major molecular-beam trends and showed nonactivated dissociative adsorption

The adsorption of H_2_-molecule is evident from an increase in the H–H bond distance (0.80 Å), which is minutely higher than the isolated H_2_-molecule, indicating that the H_2_ molecule has been activated over the Ti@Al_12_N_12_ catalyst. The dissociation of H_2_-molecule over the Ti@Al_12_N_12_ catalyst occurs through heterolytic cleavage, as shown by transition state analysis. After splitting into its atomic form (H_2_→2H^*^) through the H-dissociation process (HDR), molecular hydrogen diffuses onto the Ti metal atom and the Al metal site at the product side. The HDR needs to overcome the energy barrier of only 0.005 eV over the Ti@Al_12_N_12_ catalyst.

An increase in the H–H bond length from 0.79 Å 0.93 Å in the transition state indicates the hydrogen molecule’s activation and dissociation over the Ti@Al_12_N_12_ catalyst. The H–H bond length is an important structural parameter that significantly affects the stability of the transition state during dissociation. In the transition state, the Ti–H and Al–H interaction bond distances reduce to 1.88 Å and 1.82 Å, respectively, from their initial lengths of 2.04 Å and 1.99 Å in the intermediate state. The dissociated product (2H^*^) is more stable than the reactant molecule form (H_2_^*^), as indicated by the hydrogen dissociation reaction’s energy of −1.38 eV over the Ti@Al_12_N_12_ catalyst. The Al-H and Ti-H bond distances further decrease to 1.72 and 1.68 Å, respectively, on the product side. It is clear that the bond length of the H-H atoms in the product increased in comparison to the intermediate and transition states. The Ti@Al_12_N_12_ catalyst is excellent for hydrogen dissociation processes, and ambient conditions may overcome this activation barrier. The effectiveness of H_2_ dissociation depends on the extent of charge transfer from the metal to the antibonding molecular orbital of the H_2_ molecule and on the H_2_ molecule’s activation over the catalyst. The H–H bond is weakened by increased charge transfer from the metal to the antibonding molecular orbitals of the hydrogen molecule, thereby facilitating splitting. The extent of hydrogen molecule activation over the catalyst is the second factor.

As per the Hammond Postulate, when the bond length of H–H extends beyond its typical value of 0.74 Å upon interaction with a catalyst, it implies that the catalyst effectively promotes hydrogen activation, thereby lowering the energy barrier for bond dissociation. The Ti@Al_12_N_12_ catalyst has the smallest E_a_ value which is greater than in the isolated form. This implies that the catalyst has been activated by the hydrogen molecule.

**FIGURE 7:**
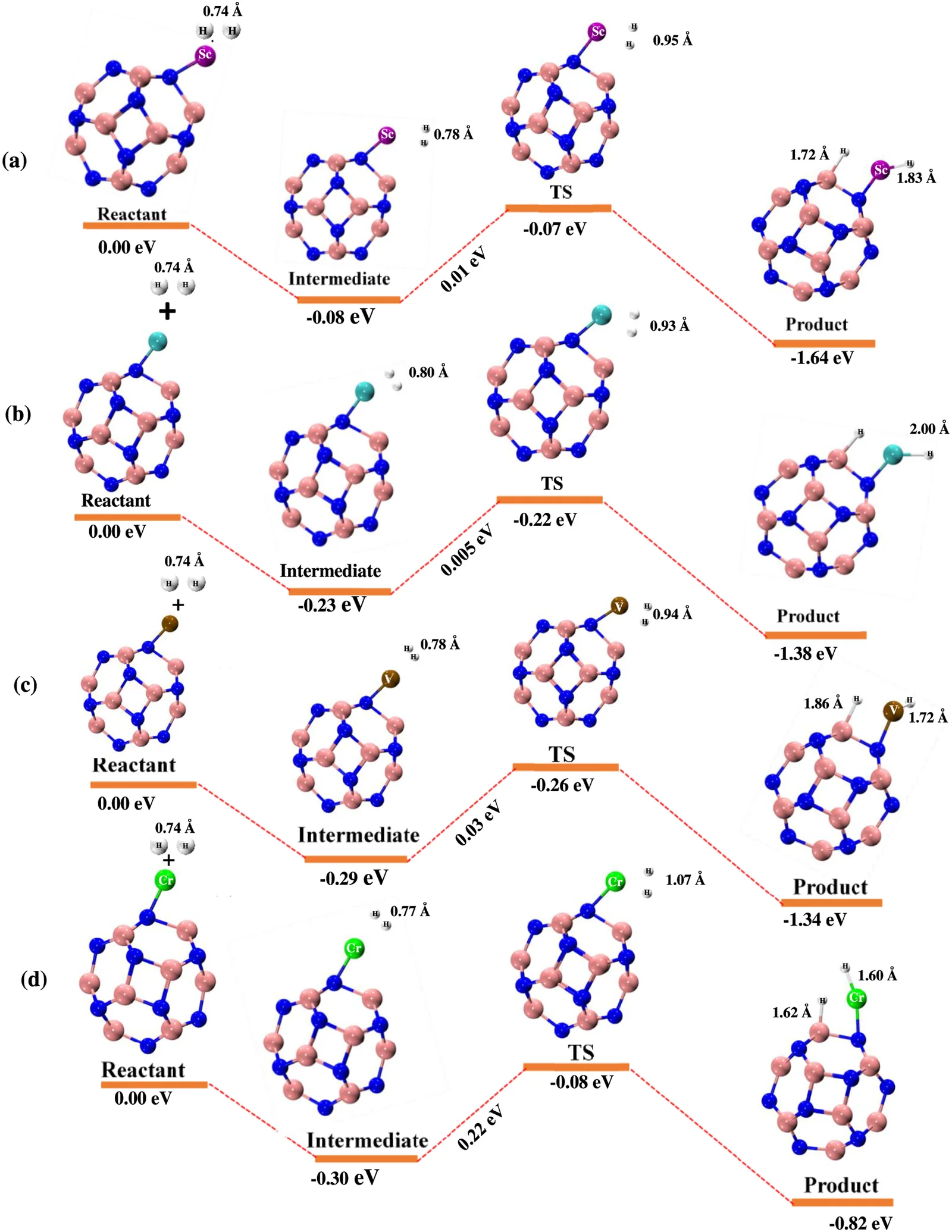
The schematic energy profile for reactant, transition state and product for **(a)** Sc@Al_12_N_12_ **(b)** Ti@Al_12_N_12_ **(c)** V@Al_12_N_12_ **(d)** Cr@Al_12_N_12_ catalyst for hydrogen dissociation reaction.

Charge transfer during the H_2_-dissociation process is the other factor. With a low activation barrier, the Ti@Al_12_N_12_ catalyst facilitates H_2_-dissociation by transferring the charge from the Al (1.62|e|) and Ti (0.45|e|) atoms of the catalyst to the hydrogen molecule’s antibonding molecular orbital. Titanium and aluminum have a significant interaction with their respective hydrogen atoms as a result of this charge transfer. In short, the strong charge transfer and enhanced hydrogen molecule activation of the Ti@Al_12_N_12_ catalyst result in a low activation barrier. The activation barrier and reaction energy values are summarized in Table 4. The schematic energy profile for the reactant, transition state and product for Sc, Ti-, V-, and Cr decorated Al_12_N_12_ are given in Figures 7a–d, while the rest are given in Supplementary Figure S1.

Similarly, to establish unequivocally that the identified transition states connect the intended reactant and product states, bidirectional intrinsic reaction coordinate (IRC) calculations are performed for the four most promising catalysts, Sc@Al_12_N_12_, Ti@Al_12_N_12_, V@Al_12_N_12_, and Cr@Al_12_N_12_, at the same level of theory used for the transition-state calculations. As shown in Supplementary Figure S2, each IRC profile displays a single energy maximum near the transition-state geometry and descends smoothly in both directions toward the molecularly adsorbed H_2_ reactant and dissociated-hydrogen product configurations. The activation barriers obtained from the IRC-validated stationary points are 0.01, 0.005, 0.032, and 0.22 eV for Sc@Al_12_N_12_, Ti@Al_12_N_12_, V@Al_12_N_12_, and Cr@Al_12_N_12_, respectively. The corresponding reaction energies are −1.545, −1.386, −1.346, and −0.824 eV, demonstrating that H_2_ dissociation is exothermic on all four catalysts. These results provide direct confirmation, through reaction-path energetics, structure, and vibration, that the reported transition states connect the intended reactants and products.

### 3.11 NBO analysis

Natural Bond Orbital (NBO) analysis was performed to evaluate the activation and dissociation behavior of the H_2_-molecule on the proposed catalyst surfaces. The NBO analysis is used to estimate the charge transfer from the d-orbitals of TM metals upon adsorption of the H_2_ Molecule. Table 6 presents the calculated NBO charges on the TM atoms of the Al_12_N_12_ nanocage, along with those on the hydrogen atom.

**TABLE 6:** Natural bond orbital (NBO) analysis of the charge distributed across the transition metal, the hydrogen atom interaction with the Al side (H_1_) and hydrogen atom interacting with the transition metal side (H_2_), and the aluminum atom in all the H_2_TM@Al_12_N_12_ complexes at transition state.

Complexes	TM |e|	H_1_ (Al side) |e|	H_2_ (TM side) |e|	Al |e|
H_2_Sc@Al_12_N_12_	0.71	−0.07	−0.18	1.59
H_2_Ti@Al_12_N_12_	0.45	−0.02	−0.10	1.62
H_2_V@Al_12_N_12_	0.40	−0.01	−0.08	1.65
H_2_Cr@Al_12_N_12_	0.47	−0.04	−0.14	1.67
H_2_Co@Al_12_N_12_	0.34	−0.08	−0.09	1.73
H_2_Ni@Al_12_N_12_	0.33	−0.02	−0.09	1.71
H_2_Cu@Al_12_N_12_	0.39	−0.04	−0.09	1.70

In all TM-metal-decorated complexes, the TM metals and Al atoms of the Al_12_N_12_ nanocage carry positive charges, while the hydrogen atoms exhibit negative charges. This charge distribution indicates that electron transfer occurs toward the hydrogen atoms during dissociation.

The H_2_-dissociation of molecular hydrogen into atomic form is facilitated by this charge transfer. The maximum charge transfer (+0.71|e|) can be observed in the catalyst Sc@Al_12_N_12._The NBO analysis of the Ti@Al_12_N_12_ catalyst is thoroughly examined, revealing insightful information. In the transition state, the approaching H atom had a −0.10|e| charge value, while the Ti metal had a charge value of +0.45|e|. In the same way, the corresponding hydrogen atom exhibited a charge of −0.02 |e|, but the Al atom had a charge of +1.62 |e|.

The fact that both metals have positive charges suggests that they are electropositive. Furthermore, the charge transfer reveals strong contact between the hydrogen molecules and metal atoms. The hydrogen molecules’ antibonding orbitals receive this charge transfer, making it easier for the molecules to split and increasing the H–H bond length in the transition state.

### 3.12 EDD analysis

**FIGURE 8:**
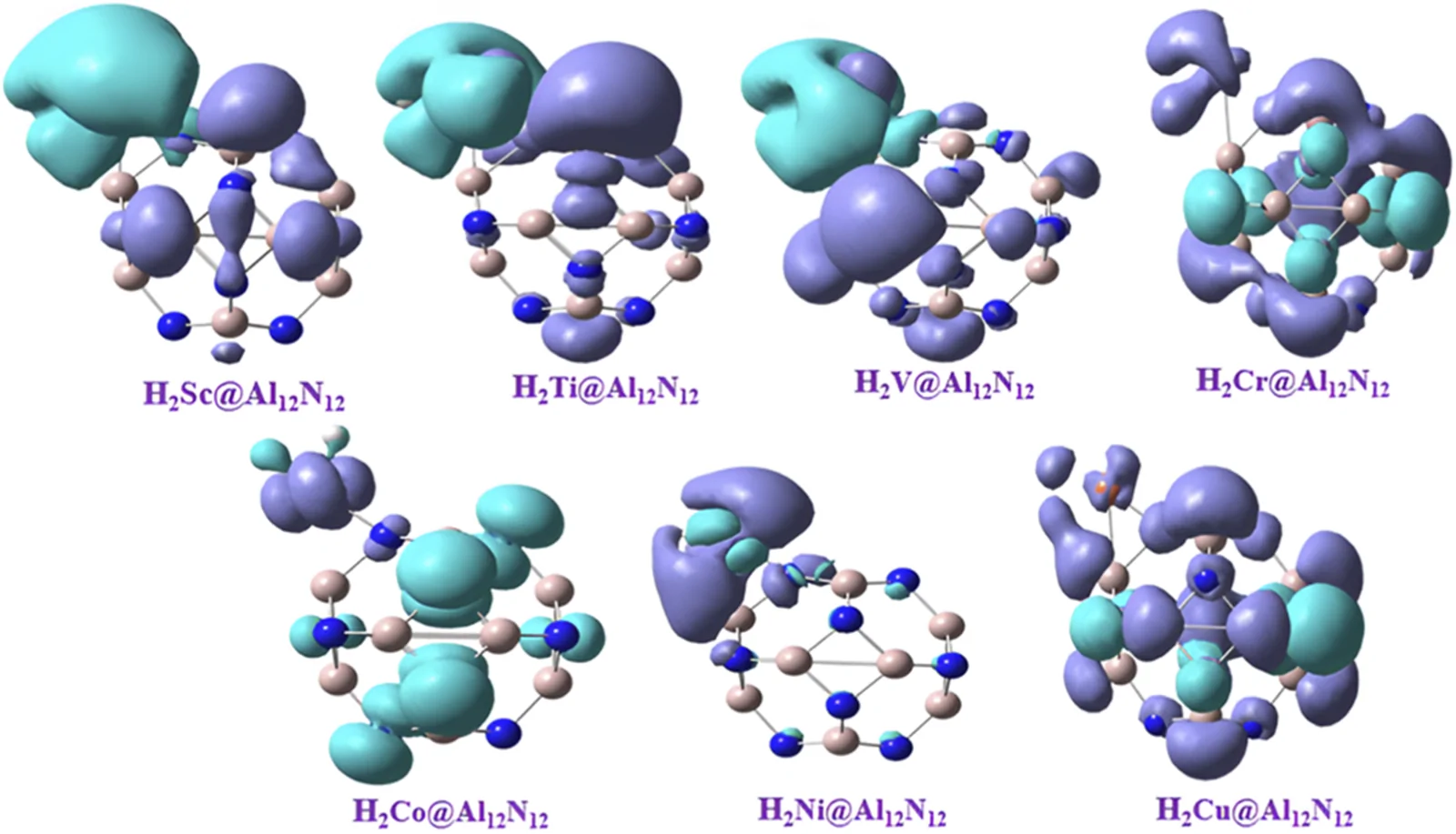
EDD analysis of H_2_TM@Al_12_N_12_ complexes.

To investigate orbital interactions and validate charge redistribution between the adsorbent and adsorbate, Electron Density Difference (EDD) analysis was performed, as shown in Figure 8. This technique illustrates the interaction zones and overlapping orbitals between atoms (Gul et al., 2023). The simultaneous presence of both colors between interacting atoms across all complexes reflects strong orbital overlap and considerable charge migration. The EDD plots for H_2_ adsorption on TM-decorated Al_12_N_12_ nanocages (TM = Sc, Ti, V, Cr, Co, Ni, Cu) provide key information regarding charge redistribution during the dissociation of hydrogen molecules and are given in Figure 8. In these visualizations, cyan regions denote zones of electron accumulation, while purple regions correspond to areas of electron depletion. The unique iso-surfaces observed between hydrogen atoms and TM centers indicate substantial orbital overlap, signifying strong interactions and efficient charge transfer. Specifically, in H_2_@Sc@Al_12_N_12_ and H_2_@Ti@Al_12_N_12_ systems, marked electron accumulation near hydrogen and depletion at the metal site demonstrate significant electron donation from the metal to the H–H bond, thereby promoting its activation and dissociation. Likewise, for H_2_@V@Al_12_N_12_ and H_2_@Cr@Al_12_N_12_, the appearance of extensive and well-defined electron accumulation and depletion regions between the TM center and hydrogen indicates effective orbital hybridization and strong catalytic engagement.

Conversely, the EDD profiles for H_2_@Co@Al_12_N_12_ and H_2_@Ni@Al_12_N_12_ exhibit comparatively moderate charge redistribution, suggesting less pronounced yet still favorable interaction mechanisms. n the case of H_2_@Cu@Al_12_N_12_, there is evident electron accumulation around the hydrogen atoms, though the interaction appears somewhat less delocalized compared to those involving early transition metals, suggesting potential differences in activation efficiency within the series. Collectively, the EDD maps demonstrate that TM doping markedly strengthens H_2_ adsorption on the Al_12_N_12_ nanocage by enabling electron transfer into the H_2_ σ* antibonding orbital, which leads to H–H bond weakening and promotes molecular dissociation.

### 3.13 IRI analysis

To determine whether covalent or non-covalent interactions exist in real space, interaction region indicator (IRI) analysis is carried out based on the electron density and its gradient in H_2_TM@ Al_12_N_12_ complexes. For covalent interactions, the electron density must be higher than 0.02 a. u.

**FIGURE 9:**
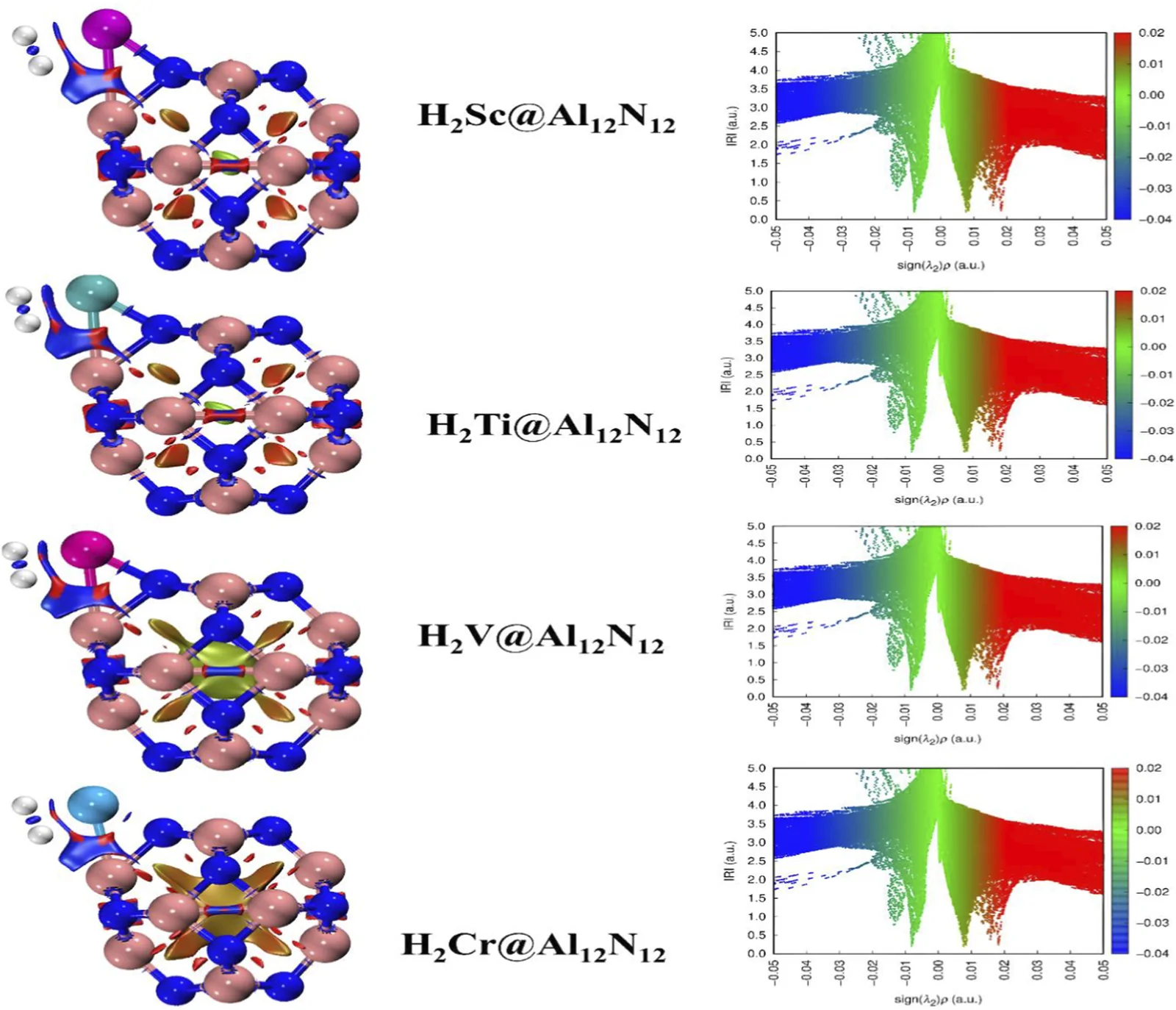
IRI analysis of H_2_Sc@Al_12_N_12_, H_2_Ti@Al_12_N_12_, H_2_V@Al_12_N_12_ and H_2_Cr@Al_12_N_12_ complexes.

The IRI function is represented on the y-axis, and the (sinλ2) ρ function is plotted on the x-axis. The presence of blue patches between the H_2_ and TM@Al_12_N_12_ catalysts indicates that they have strong interactions. Blue patches further confirm the presence of high electron density between H_2_ and TMs due to strong covalent bonding. Covalent contact spikes in IRI graphs support the concept that the TM-H and Al-H have the most effective covalent interactions for catalytic reactions. And are given in Figure 9 (from Sc to Cr) while remaining are given in Supplementary Figure S3 in Supplementary Material.

Strong or covalent interactions between the catalyst and the analyte promote the breakdown of H_2_ molecules into active H* atoms. Additionally, the presence of shared shell interactions (steric strain) is supported by the observation of the red patches on the 3D isosurface.

## 4 Conclusion

This study investigated the catalytic potential of transition metal-decorated aluminum nitride (TM@Al_12_N_12_) nanocages as single-atom catalysts (SACs) for hydrogen (H_2_) adsorption and dissociation using first-principles density functional theory (DFT) simulations. Our findings show that all transition metal-decorated Al_12_N_12_ complexes exhibit strong thermodynamic stability, with Ti@Al_12_N_12_ showing the highest interaction energy of −2.56 eV. Electron transfer from transition metals to the Al_12_N_12_ framework, as highlighted by natural bond orbital (NBO) analysis, further enhances the stability of these catalysts. Frontier molecular orbital (FMO) analysis suggests that transition-metal doping significantly reduces the HOMO-LUMO gap, indicating improved electronic properties for catalysis. The analysis of hydrogen adsorption reveals elongation of the H-H bond, signifying effective activation of H_2_ on the SACs. The activation energy for hydrogen dissociation was lowest for Ti@Al_12_N_12_, which requires only a modest barrier of 0.005 eV, confirming its efficiency as a catalyst for hydrogen dissociation. The formation of atomic hydrogen (2H*) is favored due to its greater thermodynamic stability, as the dissociation process is exothermic, releasing up to −1.64 eV. The Electron Density Difference (EDD) and NBO analyses provide further insight into the charge-transfer mechanisms that facilitate hydrogen activation and dissociation. The QTAIM and IRI analyses reveal partial covalent interactions between the transition metal atoms and hydrogen during the reaction, indicating that these complexes possess optimal catalytic characteristics for hydrogen dissociation. Overall, this study offers a comprehensive understanding of transition metal-decorated Al_12_N_12_ nanocages as promising candidates for efficient hydrogen dissociation catalysis. The insights gained from this work lay a strong foundation for the design of novel, cost-effective single-atom catalysts, enabling more efficient hydrogen energy applications in the future.

Materials used for hydrogen storage should absorb and release hydrogen easily without requiring extreme heat. Studying the transition state where the H-H bond splits easily allows control over chemical vs. physical adsorption, essential for designing materials that absorb and release hydrogen on demand. Moreover, TM@Al_12_N_12_ complexes can have catalytic efficiencies that exceed noble metals at a fraction of the cost. Quantifying the exact charge transfer helps to develop fast-response and ultra-sensitive electrochemical sensors for hydrogen leak detection in industries. Proving that synergistic dual active sites can accept charge into the H_2_ antibonding orbitals opens the door to low-temperature, low-pressure green chemistry alternatives. However, it has some limitations, as precise doping of transition metals into specific sites is exceptionally difficult. First-row transition atoms can form strong, stable hydrides that make hydrogen recombination and release kinetically unfavorable. Standard DFT functionals also struggle to accurately capture non-covalent interactions, van der Waals forces, and the highly delocalized orbital characteristics of transition metals, leading to underestimation or overestimation of the true transition-state energy barriers. Moreover, computational models also ignore factors such as solvent effects, gas pressure variations, and support interactions that exist in practical devices, presenting an “idealized” scenario of the dissociation process.

## Data Availability

The raw data supporting the conclusions of this article will be made available by the authors, without undue reservation.
