# Supplementary material for: Exploring the catalytic potential of transition metal decorated Al12N12 nanocages as a single atom catalyst for efficient hydrogen adsorption and dissociation reaction
Source: Front Chem. 2026 Sep 3;14:1879125. doi: 10.3389/fchem.2026.1879125 (PMC13582486; doi:10.3389/fchem.2026.1879125)
Supplement: Supplementary file 1 [file DataSheet1.docx]

**Exploring the Catalytic Potential of Transition Metal Decorated Al_12_N_12_ Nanocages as a Single Atom Catalyst for Efficient Hydrogen Adsorption and Dissociation Reaction**

Ali Hussain,*^a^*^,^ Aaiza Saif,^b^ Abrar Qadir,^c^ Fizza Anjum,^b^ Hamna Sharif,^b^ Muhammad Sajjad,^a,*^Abdur Rauf,*^d^* Mazhar Amjad Gilani,*^e^* Junaid Yaqoob *^b^* ^*^

*^a^Department of Chemical and Environmental Engineering, Faculty of Science and Engineering, University of Nottingham, Ningbo 315100, China*

^b^*Department of Chemistry, University of Okara, Okara 56300, Pakistan*

^c^*School of Chemistry and Molecular Engineering East China University of Science and Technology, Shanghai, China, 200237*

*^d^Department of Chemistry, University of Sahiwal, Sahiwal, 57000, Pakistan*

*^e^Department of Chemistry, COMSATS University Islamabad, Lahore Campus, Lahore 54600, Pakistan*

*** Corresponding authors E-mail addresses:**

- **Dr. Muhammad Sajjad**

Email: muhammad.sajjad@nottingham.edu.cn

- **Dr. Junaid Yaqoob**

Email: [dr.jyaqoob@gmail.com](mailto:dr.jyaqoob@gmail.com) ; [junaid.yaqoob@uo.edu.pk](mailto:junaid.yaqoob@uo.edu.pk)

**(a)**

**(b)**

**(c)**

**Figure S1:** The schematic energy profile for reactants , transition state and product for a) Co@Al_12_N_12_ b) ) Ni@Al_12_N_12_ c ) ) Cu@Al_12_N_12_ catalyst for hydrogen dissociation reaction

**Figure S2:** The The IRC profiles of the four most promising catalysts, Sc@Al₁₂N₁₂, Ti@Al₁₂N₁₂, V@Al₁₂N₁₂, and Cr@Al₁₂N₁₂ catalyst for hydrogen dissociation reaction

**Figure S3:** IRI analysis of H2TM@Al_12_N_12_ complexes

Figure S5 :Total-energy evolution of Sc@Al₁₂N₁₂ and Ti@Al₁₂N₁₂ during approximately 5.2 ps AIMD simulations.

Table S1: Calculated interaction energies (E_int_ in unit of eV ) at mixed/split basis sets of TM decorated Al_12_N_12_ nanocage

| **Complex** | **E_int_** at  6-31+G(d,p) | **E_int_** at  (def2-SVP/6-31+G(d,p) |  |
| --- | --- | --- | --- |
| [**Sc@Al_12_N_12_**](mailto:Sc@Al12N12) | -2.37 | -1.58 |  |
| [**Ti@Al_12_N_12_**](mailto:Sc@Al12N12) | -2.56 | -1.45 |  |
| [**V@Al_12_N_12_**](mailto:Sc@Al12N12) | -2.31 | -1.53 |  |
| [**Cr@Al_12_N_12_**](mailto:Sc@Al12N12) | -2.41 | -1.56 |  |
| [**Mn@Al_12_N_12_**](mailto:Sc@Al12N12) | -1.23 | -0.4 |  |
| [**Fe@Al_12_N_12_**](mailto:Sc@Al12N12) | -2.07 | -1.03 |  |
| [**Co@Al_12_N_12_**](mailto:Sc@Al12N12) | -1.69 | -1.01 |  |
| [**Ni@Al_12_N_12_**](mailto:Sc@Al12N12) | -1.63 | -1.21 |  |
| [**Cu@Al_12_N_12_**](mailto:Sc@Al12N12) | -1.29 | -0.95 |  |
| [**Zn@Al_12_N_12_**](mailto:Sc@Al12N12) | -0.38 | 0.25 |  |
